# Outcomes and Management of Dermatologic Toxicity Following Enfortumab Vedotin Rechallenge in Patients With Metastatic Urothelial Carcinoma: A Retrospective Single‐Center Study

**DOI:** 10.1002/cnr2.70466

**Published:** 2026-01-23

**Authors:** Takashi Kawahara, Yoshiyuki Nagumo, Akane Yamaguchi, Kazuki Hamada, Kozaburo Tanuma, Satoshi Nitta, Masanobu Shiga, Atsushi Ikeda, Shuya Kandori, Akio Hoshi, Hiroyuki Nishiyama

**Affiliations:** ^1^ Department of Urology, Faculty of Medicine University of Tsukuba Tsukuba Ibaraki Japan

**Keywords:** dermatologic adverse events, enfortumab vedotin, inpatient management, metastatic urothelial carcinoma, real‐world data, rechallenge

## Abstract

**Background:**

Enfortumab vedotin (EV) has demonstrated clinical benefits as a third‐line monotherapy after chemotherapy and immune checkpoint inhibitors and is currently established, in combination with pembrolizumab, as a first‐line standard therapy for metastatic urothelial carcinoma (mUC). However, dermatologic adverse events (DAEs) are common and may necessitate treatment interruption. While EV rechallenge is often considered, evidence regarding the recurrence and management of DAEs after rechallenge is limited.

**Aims:**

To analyze the incidence, timing, and severity of DAEs following EV rechallenge and assess the impact of dose modifications and initial inpatient versus outpatient management on clinical outcomes.

**Methods and Results:**

This retrospective observational study included patients with mUC who received EV at a single institution between January 2022 and December 2024. Of the 48 patients treated with EV, 36 (75.0%) developed DAEs, with a median onset of 11 days. Twenty patients resumed EV after interruption due to DAEs; 13 (65%) experienced recurrence. Recurrent DAEs tended to be of lower grade than the initial events and to be similar to the initial episode in terms of the type of DAEs. Early‐onset (≤ 7 days) DAEs were more likely to progress to grade ≥ 3. Although inpatient management enabled earlier detection and intervention, it did not reduce the incidence of severe toxicity compared to outpatient care.

**Conclusion:**

DAEs frequently recur after EV rechallenge but are typically less severe than the initial episode. Early‐onset DAEs may predict severe toxicity, underscoring the need for close monitoring and proactive management. Prospective studies are warranted to optimize rechallenge strategies and improve the safety of EV therapy.

## 1 Introduction

The first‐line treatment for metastatic urothelial carcinoma (mUC) has historically comprised platinum‐based chemotherapy, typically gemcitabine combined with cisplatin or carboplatin [1], followed by maintenance avelumab in patients showing disease control [2]. Immune checkpoint inhibitors (ICIs), such as pembrolizumab, are used as a standard second‐line therapy [3], and enfortumab vedotin (EV), an antibody–drug conjugate targeting Nectin‐4, is the standard third‐line option [4]. Recently, the phase 3 EV‐302/KEYNOTE‐A39 trial demonstrated that combination therapy of EV plus pembrolizumab significantly improved survival compared with platinum‐based chemotherapy [5], establishing it as a new global first‐line standard.

The significant clinical efficacy of EV has been demonstrated; however, its use is frequently complicated by dermatological adverse events (DAEs), which are the most common treatment‐related toxicities. In clinical trials, DAEs were observed in 47% of patients receiving third‐line EV monotherapy [4], with grade ≥ 3 events occurring in 14.5%, whereas in the first‐line EV plus pembrolizumab combination therapy, DAEs occurred in 66.8% of patients, including 15.5% with grade ≥ 3 severity [5]. Notably, emerging real‐world data suggest a higher incidence and severity of EV‐associated DAEs in Japanese patients than in global cohorts. A multicenter retrospective study reported that the dermatologic toxicity incidence exceeded 50% [6], and a large‐scale real‐world analysis by Kita et al. confirmed the high incidence and identified specific risk factors associated with severe cutaneous toxicity in this population [7].

DAEs are common during EV treatment and have recently been suggested as potential prognostic markers for improved survival in patients with mUC [7, 8, 9]. Despite the clinical importance of DAEs, evidence regarding effective management approaches is lacking. In particular, the safety and outcomes of rechallenging EV after treatment interruption owing to DAEs remain largely unexplored. The recurrence rate, severity, and timing of DAEs upon re‐administration and the potential role of early inpatient monitoring in mitigating toxicity remain unclear. To clarify these issues, we conducted a retrospective observational study to evaluate the recurrence and severity of DAEs following EV rechallenge in patients with mUC. We aimed to evaluate the safety and outcomes of EV rechallenge after treatment interruption due to DAEs and assess whether dose adjustment and early inpatient management could support safe and effective treatment continuation.

To this end, we retrospectively analyzed patients with mUC treated with EV at our institution. Our objectives were to investigate the recurrence rate and severity of DAEs following EV rechallenge after initial toxicity and investigate the impact of dose modification and the effect of early inpatient management on treatment outcomes.

## 2 Methods

### 2.1 Study Design and Patient Population

This retrospective observational study was conducted at University of Tsukuba Hospital between January 1, 2022, and December 31, 2024 (IRB Approval No. 05‐280). An opt‐out consent form was used to obtain informed consent. Patients with mUC who received EV following chemotherapy and/or ICIs treatment were included. The inclusion criteria included histologically or radiographically confirmed unresectable or mUC, EV administration, and available clinical data. Patients with incomplete medical records were excluded. EV was initiated at the standard recommended dose of 1.25 mg/kg on days 1, 8, and 15 of a 28‐day cycle. Treatment was withheld for grade ≥ 2 DAEs. Treatment continuation or treatment rechallenge was permitted once the toxicity improved to grade ≤ 1. Dose reduction was performed at the discretion of the attending physician, following the same criteria as those in the pivotal clinical protocol: from 1.25 to 1.00 mg/kg and from 1.00 to 0.75 mg/kg in cases with Grade 2–3 adverse events. Among patients who developed DAEs, when the initial DAE was of Grade 1, treatment continuation or temporary interruption was considered in consultation with the attending physician and/or a dermatologist. For patients who developed Grade 2 or higher DAEs, EV was withheld until improvement to Grade 1 or lower before rechallenge.

### 2.2 Data Collection and Outcomes

Demographic and clinical characteristics, including age, sex, Eastern Cooperative Oncology Group (ECOG) performance status (PS), primary tumor site, histologic type at initial diagnosis, sites of metastasis at initiation of EV therapy, previous systemic therapies, and prior ICI exposure, were retrieved from electronic medical records. Key outcomes included the frequency, timing, and severity of EV‐induced DAEs. DAE severity was evaluated according to the *Common Terminology Criteria for Adverse Events*, version 5.0. We assessed the following management strategies: inpatient versus outpatient care, treatment continuation or interruption, and the recurrence or worsening of DAEs after EV rechallenge. The decision between inpatient and outpatient management was made based on the discussion between the patient and the attending physician. Patients who preferred inpatient management were hospitalized until day 15 of the first cycle, and subsequent cycles were administered in an outpatient setting. When Grade 2 or 3 adverse events occurred, rechallenge after recovery was recommended under inpatient or outpatient management, following discussion with the patient.

### 2.3 Statistical Analysis

Categorical and continuous variables were analyzed using Fisher's exact test and the Mann–Whitney *U*‐test, respectively. Survival was estimated using the Kaplan–Meier method. Statistical significance was set at *p* < 0.05. All analyses were performed using Prism (v10). A swimmer plot was created using the R package (ver. 4.0.2; R Foundation).

## 3 Results

### 3.1 Patient Characteristics

Patients' characteristics are shown in Table 1. In total, 79% of the patients were male, and the median age was 72 years (range, 41–85 years). The median follow‐up period was 222 (range, 14–1120) days. ECOG PS was 0–1 in 96% and 2 in 4.2%. No patient had skin comorbidities. The primary tumor sites included the upper urinary tract (19%), the bladder (67%), and both (15%). Median EV treatment cycle was four (range, 1–15).

**TABLE 1:** Patient characteristics.

Characteristic	*n* (%) or median (range)
Patient number	48
Median follow‐up time, day (range)	222 (14–1120)
Median age, year (range)	72 (41–85)
Sex	
Male	38 (79%)
Female	10 (21%)
ECOG performance status	
0	30 (63%)
1	16 (33%)
2	2 (4.2%)
Origin site of primary disease—no (%)	
Upper urinary tract	9 (19%)
Bladder	32 (67%)
Both	7 (15%)
Histologic type at initial diagnosis	
Urothelial carcinoma	40 (83%)
Urothelial carcinoma with mixed type	3 (6.3%)
Others	2 (4.2%)
Unknown	3 (6.3%)
Sites of metastasis at initiation of EV therapy	
Lymph node only	11 (23%)
Visceral site	27 (56%)
Liver	11 (23%)
Previous systemic therapies	
1	4 (8.3%)
2	42 (88%)
3 ≦	2 (4.2%)
Prior immune checkpoint inhibitors exposure	
Yes	44 (92%)
No	4 (8%)
Treatment setting	
Inpatient	36 (75%)
Outpatient	12 (25%)
Median EV cycles (range)	4 (1–15)

### 3.2 Incidence, Severity, and Timing of DAEs

**FIGURE 1:**
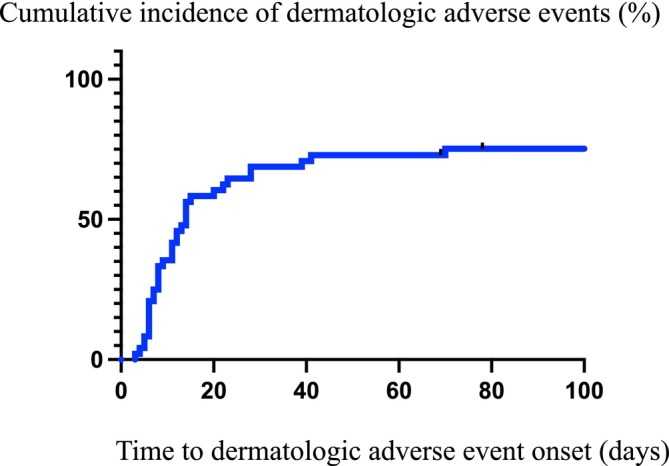
Cumulative incidence of dermatologic adverse events (DAEs) following initiation of enfortumab vedotin (EV) therapy. Kaplan–Meier analysis for the time to onset of DAEs in patients treated with EV. The *x*‐axis represents the number of days from the first EV administration to the onset of DAEs, and the *y*‐axis represents the cumulative incidence. The median time to DAE onset was 11 days (range: 3–70 days), with most events occurring within the first 30 days of treatment. Information on patients without DAE was censored at the final follow‐up visit.

Among the 48 patients treated with EV, 36 (75.0%) developed DAEs. Characteristics of patients with or without DAE are shown in Table 2. Baseline patient characteristics did not differ between the groups with and without DAEs. The DAE grade at initial onset was as follows: 1 in 23 patients, 2 in 10 patients, and 3 in 3 patients. The median time to DAE onset was 11 days (range, 3–70 days), with most events occurring within the first 30 days of treatment (Figure 1).

**TABLE 2:** Patient characteristics with or without DAEs.

Characteristic	DAE		*p*
	Present	Absent	
Patient number	36	12	
Median age, year (range)	72 (57–85)	73 (41–84)	0.86^a^
Sex			
Male	28	10	> 0.99^b^
Female	8	2	
ECOG performance status			
0	23	7	0.60^b^
1	12	4	
2	1	1	
Origin site of primary disease			
Upper urinary tract	8	1	0.61^b^
Bladder	23	9	
Both	5	2	
Previous systemic therapies			
1	3	1	0.75^b^
2	32	10	
3 ≦	1	1	
Prior immune checkpoint inhibitors exposure			
Yes	33	11	> 0.99^b^
No	3	1	
Initial treatment setting			
Inpatient	29	6	0.061^b^
Outpatient	7	6	

^a^ Mann–Whitney *U* test.

^b^ Chi‐square test.

**FIGURE 2:**
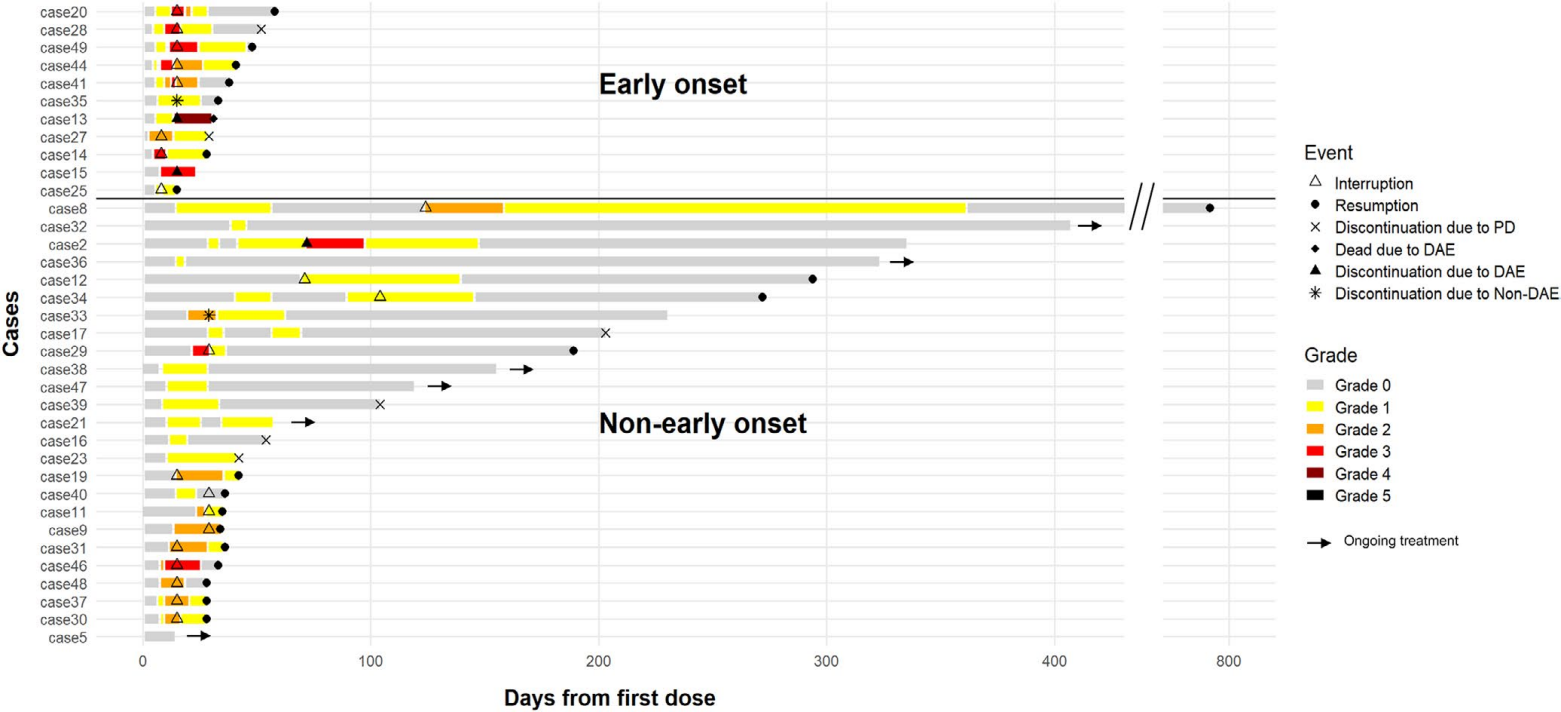
Timing and severity of DAEs after initiation of EV therapy. Swimmer plot showing the timing, severity, and clinical course of DAEs after the initiation of EV. Early‐onset DAEs, defined as those occurring within 7 days after the first EV dose, were observed in 12 patients (25%), while non‐early‐onset DAEs, defined as those occurring on or after day 8, were observed in 24 patients (50%). Bars represent the longitudinal clinical course for each patient, with colors indicating the grade of DAEs according to *Common Terminology Criteria for Adverse Events*. Symbols denote key clinical events: Triangles indicate treatment interruption, closed circles indicate treatment resumption, crosses indicate discontinuation due to progressive disease (PD), diamonds indicate death due to DAEs, triangles with black fill indicate discontinuation due to DAEs, and asterisks indicate discontinuation due to non‐DAE causes. Arrowheads indicate ongoing treatment at the last follow‐up. Among patients with early‐onset DAEs, several showed progression to higher‐grade toxicity, including Grade 3 and Grade 5 events. In contrast, most non‐early‐onset DAEs were initially low grade, although some patients experienced subsequent grade progression during continued EV administration.

The types and frequencies of DAEs associated with EV are shown in Table 3. Among DAEs, rash maculopapular was the most frequently observed (*n* = 28), followed by bullous dermatosis (*n* = 3). A single case of toxic epidermal necrolysis (TEN) was observed. Early‐onset DAEs—defined as those occurring within 7 days after first EV initiation—were observed in 12 patients (25%). Initial DAEs were of Grade 1 in 8 patients, Grade 2 in 1 patient, and Grade 3 in 3 patients. Among initial DAEs of Grade 1 or 2, 4 patients progressed to Grade 3 and 1 progressed to Grade 5 toxicity (Figure 2). Non‐early‐onset DAEs—defined as those occurring on or after day 8 following first EV initiation—were observed in 24 patients (50%). Initial DAEs were of Grade 1 in 14 patients, Grade 2 in 9 patients, and Grade 3 in 1 patient. One patient showed DAE progression from Grade 2 to 3 during first cycle, and one patient showed DAE progression from Grade 1 to 3 after three cycles of EV administration (Figure 2).

**TABLE 3:** Types and frequencies of dermatologic adverse events associated with EV.

Type of dermatologic adverse event	*n* (%)
Rash maculopapular	28 (58%)
Bullous dermatosis	3 (6.3%)
Eczema	2 (4.2%)
Toxic epidermal necrolysis	1 (2.0%)
Dry desquamation	1 (2.0%)
Pruritus	1 (2.0%)

### 3.3 Clinical Course After Initial Onset of DAEs

**FIGURE 3:**
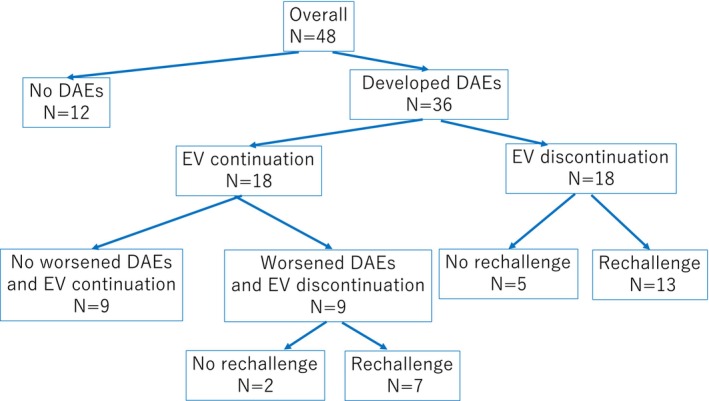
Clinical course of patients treated with EV according to the development and management of DAEs. The flow diagram illustrates the overall clinical course of 48 patients treated with EV for mUC. Among them, 36 (75.0%) developed DAEs, while 12 (25.0%) did not. Of the 36 patients who developed DAEs, 18 patients continued EV therapy after onset, and therapy was interrupted in 18 patients upon DAE onset. Among patients who continued EV therapy, nine continued without worsening of DAEs, whereas the remaining nine patients eventually discontinued EV treatment owing to worsening DAEs. Among the nine patients who discontinued the treatment, seven were rechallenged after DAEs improved. Eighteen patients temporarily discontinued EV after DAE onset, and 13 patients were rechallenged with EV therapy after DAEs improved. Subsequent rechallenge was performed in 20 patients (74.1%) following either continuation or interruption.

Of the 36 patients who developed DAEs, 18 continued to receive EV therapy because the DAE was of Grade 1. Among patients who continued EV therapy, nine continued treatments without worsening of DAEs, whereas the remaining nine eventually discontinued EV treatment owing to worsening of DAEs. Among the nine patients who discontinued treatment, seven were rechallenged with EV therapy after DAEs had improved. The other 18 patients temporarily discontinued EV after DAE onset; 13 patients were rechallenged with EV therapy after DAEs had improved (Figure 3). Among the 18 patients who discontinued EV following the initial onset of DAEs, 14 showed improvement after interruption, while 4 experienced worsening of DAEs despite treatment interruption. Seven did not resume EV treatment because two were due to DAEs, two were due to other adverse events, and three were due to disease progression (Figure 2).

### 3.4 Recurrence After EV Rechallenge

Among the 27 patients who discontinued EV due to DAEs, 20 resumed treatments. Among the 20 patients, 14 restarted EV after complete resolution of DAEs, and 6 restarted EV after improvement to Grade 1 (Figure 2). The median interval until treatment resumption was 23 days (range, 7–674 days). Regarding dose adjustment, all eight patients who had experienced initial Grade 3 DAEs underwent dose reduction. Among the eight patients with initial Grade 2 DAEs, six resumed EV at a reduced dose and two resumed at the same dose. Of the four patients with initial Grade 1 DAEs, two resumed EV at the reduced dose and two at the same dose.

**FIGURE 4:**
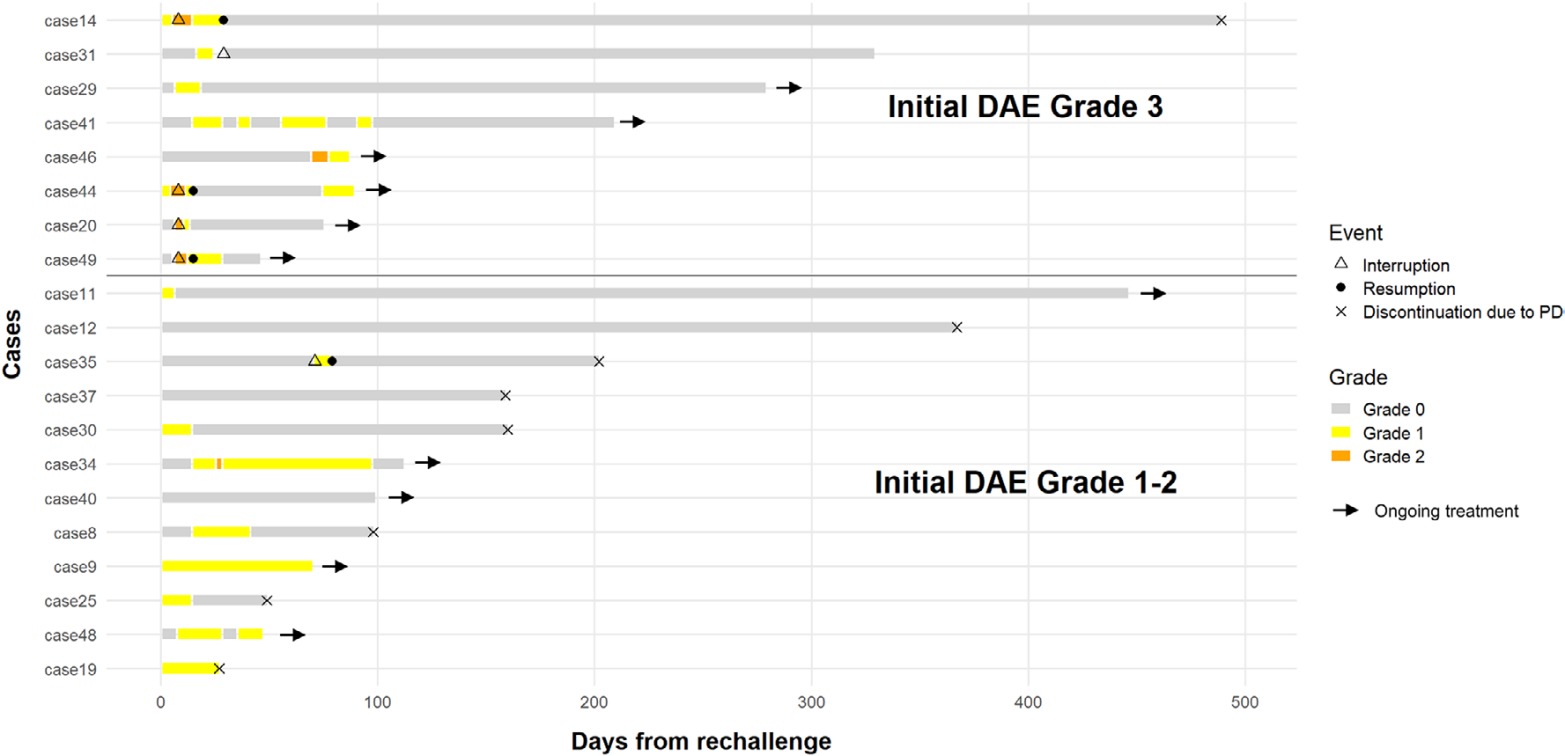
Clinical course after EV rechallenge according to initial DAE grade. Swimmer plot illustrating the clinical course of patients who resumed EV after initial DAEs, stratified by the severity of the initial DAE (upper panel: Initial Grade 3; lower panel: Initial Grade 1–2). Each horizontal bar represents the duration from EV rechallenge to the last follow‐up or treatment discontinuation. Color coding indicates the severity of DAEs during rechallenge. Triangles indicate treatment interruption, closed circles indicate treatment resumption, crosses indicate discontinuation due to disease progression, and asterisks indicate discontinuation due to non‐DAE reasons. Arrows denote ongoing treatment at the last follow‐up. Among patients with initial Grade 3 DAEs, all 8 experienced recurrent DAEs (Grade 2 in 5 and Grade 1 in 3). Of 12 patients with initial Grade 1–2 DAEs, 5 developed recurrent DAEs (one with Grade 2 and four with Grade 1). In Case 9, although no grade escalation was observed after rechallenge, a worsening within Grade 1 severity was noted. None of the 13 patients who developed recurrent DAEs discontinued EV because of DAE recurrence.

Recurrence of DAEs after treatment resumption was observed in 13 patients. Among those with initial Grade 3 DAEs, all eight experienced DAE recurrence (5 with Grade 2 and 3 with Grade 1), (Figure 4). Of eight patients with initial Grade 2 DAEs, three experienced DAE recurrence (3 with Grade 1), and of four patients with initial Grade 1 DAEs, two experienced DAE recurrence (1 each with Grade 2 and Grade 1). None of the 13 patients who developed recurrent DAEs discontinued EV owing to DAE recurrence.

Among the 20 patients who resumed treatment, 16 experienced the same type of DAEs as that during the initial event. None of the 13 patients who developed recurrent DAEs discontinued EV owing to DAE recurrence (Figure 4).

### 3.5 Impact of Inpatient Versus Outpatient Management

Among the 48 patients treated with EV, EV treatment was initiated in 36 (75%) at inpatient setting and 12 (25%) at outpatients setting. Patient characteristics are shown in Table S1. Baseline patient characteristics did not differ between the inpatient and outpatient management groups. DAEs occurred in 7 of the 12 outpatients (58%) and 29 of the 36 inpatients (81%). In the outpatient group, the initial severity was Grade 1 in 4 patients, Grade 2 in 1 patient, and Grade 3 in 2 patients, with DAEs of grade ≥ 3 observed in 2 patients (17%). One inpatient developed fatal Grade 5 toxicity. The patient developed TEN and subsequently sepsis and multiple organ failure, leading to death. In the inpatient group, the initial severity was Grade 1 in 18 patients, Grade 2 in 9 patients, and Grade 3 in 2 patients, with DAEs of grade ≥ 3 observed in 2 patients (5%) at onset. However, including subsequent progression after onset, five patients experienced worsening severity, resulting in an overall incidence of grade ≥ 3 DAEs of 17% (6/36) among inpatients. For subsequent cycles, the 12 patients who initiated EV at the outpatient setting continued treatment at outpatients. Among the 36 patients who initiated EV at inpatients setting, 3 who developed Grade 3 toxicity received the second cycle at inpatient management; 1 of these 3 patients remained hospitalized through the third cycle, after which treatment was continued at outpatient setting.

## 4 Discussion

Our findings clarify the recurrence rate and severity of DAEs upon rechallenge with EV following initial toxicity. Approximately half of the patients who resumed EV after a temporary interruption experienced recurrence of DAEs, although most recurrences were not more severe than the initial episode. Notably, our findings suggest that early‐onset DAEs—defined as those occurring within 7 days after EV initiation—are associated with a higher risk of progression to grade ≥ 3 toxicity. These results provide novel insights into the risk stratification of patients during EV therapy and highlight the importance of close monitoring, particularly in those who develop skin toxicities early during treatment.

Previous clinical trials, such as EV‐301, reported that dermatologic toxicities were among the most common adverse events associated with EV, with grade ≥ 3 events occurring in approximately 15% of patients [4]. Recent real‐world studies in Japanese populations suggest an even higher incidence of DAEs and an earlier onset than in global cohorts [6, 7]. Consistent with these observations, our study demonstrated a high frequency of DAEs (75%) and validated that most toxicities developed within the first month of treatment. However, evidence regarding the clinical course after treatment interruption due to DAEs and the outcomes of EV rechallenge is limited. Our findings provide additional real‐world data regarding the feasibility and risks associated with EV re‐administration in Japanese patients who may have a higher incidence of dermatologic toxicity. Combination therapy with EV and pembrolizumab has emerged as a new standard first‐line treatment for mUC, and the management of DAEs after rechallenge is becoming increasingly important for maintaining long‐term treatment continuity [5, 10, 11].

Recurrence of DAEs after EV rechallenge was more frequent in patients who had experienced higher‐grade toxicities during the initial episode. All 8 patients with initial Grade 3 DAEs experienced recurrence even after dose reduction, whereas recurrence was observed in 5 of the 12 patients with initial Grade 2 or Grade 1 DAEs. Only 1 patient with an initial Grade 1 DAE showed an increase to Grade 2 upon recurrence. Although recurrence of DAEs was frequent after EV rechallenge, worsening beyond the initial grade was uncommon, indicating that EV rechallenge may be feasible and safely performed once DAEs have adequately improved.

Our analysis demonstrated that the timing of DAE onset after EV treatment initiation was associated with the severity of skin toxicity. Patients who developed DAEs within 7 days of treatment initiation exhibited a higher risk of progressing to grade ≥ 3 toxicity, compared to those with later onset. In contrast, patients who experienced DAEs 14 days or later had a relatively lower risk of severe events. These findings suggest that early‐onset DAEs may reflect heightened susceptibility to severe cutaneous toxicity and underscore the importance of close monitoring, particularly during the first week of EV therapy. Early identification of high‐risk patients can provide more aggressive dermatological interventions to mitigate severe outcomes.

We assessed the impact of the initial inpatient and outpatient management during the early phases of EV therapy. Inpatient management allowed for earlier detection and intervention of DAEs, reflected as the shorter time to DAE onset and earlier initiation of supportive care, compared to outpatient management. Earlier detection reflected closer clinical observation rather than true biological differences in onset. Although inpatient monitoring enabled prompt recognition and intervention, it did not significantly prevent progression to severe DAEs. Therefore, while closer observation during inpatient care may facilitate timely management of DAEs, detection bias should be considered. Additional strategies, such as early dose modification or prophylactic dermatologic interventions, may be required to mitigate severe toxicity during EV therapy. Notably, with EV and pembrolizumab regimens, inpatient management can support timely dermatologic assessment and improve outcomes for severe cutaneous reactions [12]. In regimens involving EV, inpatient care may be beneficial for facilitating early dermatologic management and improving outcomes in patients with severe cutaneous reactions.

This study has some limitations that warrant further consideration. First, this was a retrospective observational analysis conducted at a single institution with a relatively small sample size. Second, the decision to initiate EV in the inpatient or outpatient setting was made through discussions between the patient and the treating physician, without standardized criteria. Third, an optimal duration of inpatient monitoring for effectively managing DAEs has not yet been established. As this study was conducted in a Japanese population, caution should be exercised when extrapolating these findings to other populations. Nevertheless, fundamental insights regarding early detection and rechallenge strategies are likely broadly applicable and merit further validation in diverse patient cohorts through prospective studies.

## 5 Conclusions

Our study demonstrated that DAEs recurred in more than half of the patients following EV rechallenge, although the severity was generally comparable to that of the initial episode. Early‐onset DAEs were significant risk factors for progression to grade ≥ 3 toxicity, emphasizing the necessity for vigilant monitoring and early intervention. Prospective investigations are warranted to establish optimal management strategies to ensure the safe and sustained administration of EV therapy.

## Author Contributions

Conceptualization: Takashi Kawahara, Yoshiyuki Nagumo, and Hiroyuki Nishiyama. Formal analysis: Takashi Kawahara and Akane Yamaguchi. Investigation: Kazuki Hamada. Data curation: Kozaburo Tanuma. Writing – original draft: Takashi Kawahara, Satoshi Nitta, Masanobu Shiga, and Atsushi Ikeda. Writing – review and editing: Takashi Kawahara, Shuya Kandori, and Akio Hoshi. Supervision: Hiroyuki Nishiyama All authors have read and agreed to the published version of the manuscript.

## Funding

The authors have nothing to report.

## Ethics Statement

The study was conducted in accordance with the Declaration of Helsinki and approved by the Institutional Review Board of the University of Tsukuba Hospital (Approval No. 05‐280; date of approval: July 16, 2024).

## Conflicts of Interest

The authors declare no conflicts of interest.

## Supporting information


**Table S1:** cnr270466‐sup‐0001‐Table.xlsx.

## Data Availability

No new data were created or analyzed in this study. Anonymized data are available from the corresponding author upon reasonable request, subject to IRB and privacy restrictions.
